# Stromal cell-expressed malignant gene patterns contribute to the progression of squamous cell carcinomas across different sites

**DOI:** 10.3389/fgene.2024.1342306

**Published:** 2024-07-12

**Authors:** Kaiyan Qi, Guangqi Li, Yuanjun Jiang, Xuexin Tan, Qiao Qiao

**Affiliations:** ^1^ Department of Radiation Oncology, The First Hospital of China Medical University, Shenyang, China; ^2^ Department of Biotherapy, Cancer Center and State Key Laboratory of Biotherapy, West China Hospital, Sichuan University, Chengdu, China; ^3^ Department of Urology, The First Hospital of China Medical University, Shenyang, China; ^4^ Department of Oral Maxillofacial-Head and Neck Surgery, School and Hospital of Stomatology, China Medical University, Shenyang, China

**Keywords:** squamous cell carcinoma, head and neck, stromal cell, cancer associated fibroblast, lung cancer

## Abstract

**Background:**

Squamous cell carcinomas (SCCs) across different anatomical locations possess common molecular features. Recent studies showed that stromal cells may contribute to tumor progression and metastasis of SCCs. Limited by current sequencing technology and analysis methods, it has been difficult to combine stroma expression profiles with a large number of clinical information.

**Methods:**

With the help of transfer learning on the cell line, single-cell, and bulk tumor sequencing data, we identified and validated 2 malignant gene patterns (V1 and V5) expressed by stromal cells of SCCs from head and neck (HNSCC), lung (LUSC), cervix (CESC), esophagus, and breast.

**Results:**

Pattern V5 reflected a novel malignant feature that explained the mixed signals of HNSCC molecular subtypes. Higher expression of pattern V5 was related to shorter PFI with gender and cancer-type specificity. The other stromal gene pattern V1 was associated with poor PFI in patients after surgery in all the three squamous cancer types (HNSCC *p* = 0.0055, LUSC *p* = 0.0292, CESC *p* = 0.0451). Cancer-associated fibroblasts could induce HNSCC cancer cells to express pattern V1. Adjuvant radiotherapy may weaken the effect of high V1 on recurrence and metastasis, depending on the tumor radiosensitivity.

**Conclusion:**

Considering the prognostic value of stromal gene patterns and its universality, we suggest that the genetic subtype classification of SCCs may be improved to a new system that integrates both malignant and non-malignant components.

## Background

Squamous cell carcinomas (SCCs) are among the most prevalent forms of solid cancers, originating from epithelia of multiple organs, such as head and neck, esophagus, lung, skin, and cervical. SCCs across different anatomical locations possess common histopathological and molecular features (Schwaederle et al., 2015; Chai et al., 2020), showing a high tendency to relapse and metastasize. Although there has been substantial progress in surgery, radiotherapy, and targeted therapy of these tumors, the prognosis remains stagnant due to locoregional recurrences, second primary tumors, and distant metastases.

There is increasing evidence that tumors result from disordered organ homeostasis rather than malignant single cell (or cells). A landmark study on SCCs of head and neck (HNSCC) first put forward the theory of field cancerization (Slaughter et al., 1953) to explain the surprisingly high frequency of multifocality and second primary tumors in epithelial cancers (Rahal et al., 2022). In the first step of field cancerization, genetically altered epithelial proliferate to form a field lesion without any histological changes. Then certain cells within this field got further critical changes and transformed into malignant cells, resulting in multi-foci primary tumors (changes occur at the same time) or second primary tumors (changes occur in sequence) (Baumeister et al., 2021). The theory has been accepted in numerous epithelial cancers, drawing attention to normal-appearing adjacent tissue surrounding the tumor. However, in this model, changes of the epithelium are primary determinants, while the stromal alterations received relatively less attention. As researches on cancer drivers shifting from cancer cells to tumor microenvironment (TME), alteration in tumor stroma has been found to play a primary role in carcinogenesis (Bhowmick et al., 2004; Dotto, 2014). Many cancer driver mutations can be found in tumor stroma (Guan et al., 2020), which shows different structures from normal stroma (Plava et al., 2019; Xu et al., 2022). Hypotheses have been proposed that stroma could contribute to field cancerization (Cox, 2021), and researches have shown the contribution of stromal alteration to cancer progression and metastasis (Hanahan and Weinberg, 2011; Valkenburg et al., 2018; Chhabra and Weeraratna, 2023).

Although the deconvolution technique and single-cell sequencing are under rapid development, it is still hard to illustrate cancer stromal alterations across different types of transcriptomic data. Previous researches on the relationship between the genetic features and tumor prognosis of SCCs were limited to single anatomical locations, focusing on cancer cells (or mixed signals) rather than stromal cells. One reason is that bulk sequencing loses the signal complexity within a tumor while single-cell sequencing compromises on the number of tumors sequenced, therefore it has been hard to combine stroma expression profiles with a large number of prognostic information. Therefore, previous sequencing-based studies on cancer stroma focused on stromal cell proportion rather than gene alteration of stromal cells. To solve this problem, we designed a series of analytical approaches based on matrix factorization, transfer learning and deconvolution for cell line, single-cell, and bulk tumor sequencing data. We recognized squamous malignant gene patterns across different organs and projected the patterns to specific datasets to explore the relationship between gene patterns and phenotypes. Focusing on the cancer stroma, we explored the prognostic value of the stromal-expressed malignant gene patterns and their contribution to subtype classification of SCCs.

## Methods

### Datasets

The microarray gene expression profiles of the 63 squamous cancer cell lines were selected from the Sanger Cell Line Affymetrix Gene Expression Project (GSE68950) (NCHBI, 2015). The single-cell RNA sequencing (scRNA-seq) data of head and neck squamous cancer (HNSCC) has been published previously (Puram et al., 2017). Single-cell transcriptomes for 5,902 cells from 18 HNSCC patients with both more than 2,000 detected genes and an average housekeeping expression level above 2.5 passed initial quality controls. RNA-seq, DNA methylation, and clinical data of TCGA HNSCC, lung squamous cell carcinoma (LUSC), and cervical squamous cell carcinoma (CESC) datasets were obtained from Genomic Data Commons Data Portal (National Cancer Institute, 2024). The 303 reference methylation profiles of the known cell types used for deconvolution were gathered from the Gene Expression Omnibus (GEO) (NCHBI, 2023). The microarray gene expression profiles of five HNSCC cell lines grown without and with patient-matched cancer-associated fibroblasts (CAFs) were obtained from GSE178153 (NCHBI, 2021). The 98 paired normal and HNSCC tumor samples used in Figure 3H were selected from (Smetana et al., 2020).

### Pattern identification and transfer learning

The nonnegative matrix factorization (NMF) has been applied for multiple purposes including image processing, language modeling, and genomic feature extraction (Devarajan, 2008). This approach uses a limited number of basic components to interpret the target data as accurately as possible. We performed NMF algorithm (Gaujoux and Seoighe, 2010) in a relatively heterogeneous dataset of multiple squamous cancer types to recognize patterns associated with squamous malignancy. Then we projected the patterns to specific datasets to explore the relationship between gene patterns and phenotypes. For example, we transferred the learned patterns to a single cell transcriptome to explore their distribution in different cell types. This transfer learning process was performed using the R package “projectR” (23). Pattern expression levels was defined as projection score.

### Deconvolution and cell-type gene expression estimation

Deconvolution technology for bulk tissue sequencing aims to fill the gap where bulk sequencing loses the signal complexity within a tumor while single-cell sequencing compromises on the number of tumors sequenced. In many cases where a sufficient number of patients is needed to satisfy the statistical test, deconvolution is the best way if we want to keep the sequencing accuracy at the level of cell types rather than bulk tissue.

The Edec R package (Onuchic et al., 2016) was used for deconvolution. A total of 303 GEO DNA methylation profiles of known cell types (cancer cells, stromal cells, and immune cells, Supplementary Table S1) were collected as reference (Li et al., 2021). First, we identified 400 DNA loci from the 450 k methylation profile which allowed us to accurately distinguish the three reference cell types (Supplementary Figure S1A). Based on the 400 loci, we deconvoluted the TCGA HNSCC methylation profile into three subtypes. The three parts showed a high correlation with cancer, stroma, and immune reference profiles, respectively (Supplementary Figure S1B). We validated the deconvolution result by comparing the cancer cell type proportion with the cancer purity calculated by the ABSOLUTE method (Carter et al., 2012) using somatic copy-number. The cancer proportion estimated by the two methods showed a high correlation (Cor = 0.81, *p* < 0.0001, Supplementary Figure S1C). Then we estimated the cell type gene expression through a constrained least squares fit (Onuchic et al., 2016) based on the cell type proportions obtained by deconvolution.

### Statistical analysis

All the statistical analyses were performed under R software version 4.1.0. Cox regression and survival analysis were performed using the R package ‘‘survival”. Cutoffs were identified using the R package “survminer”. Differentially expressed genes were identified using the R package “edgeR”. Gene set enrichment analysis was performed using the online tool Metascape (Zhou et al., 2019).

## Results

### Identification of six squamous malignant patterns across multiple cancer types

**FIGURE 1:**
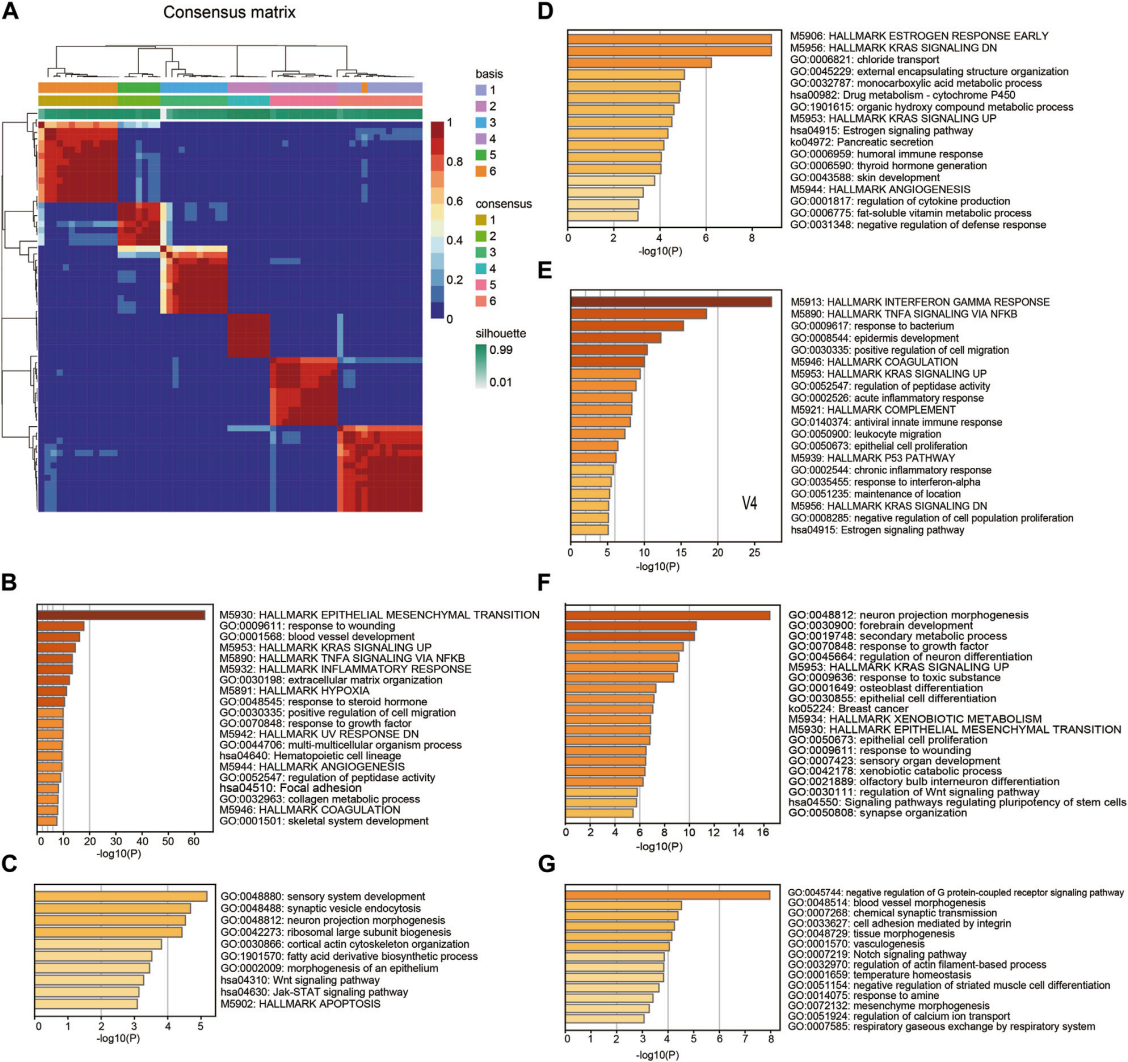
Pattern recognition and functional annotation. **(A)** Consensus matrix showing the 6 patterns identified by NMF. **(B-G)** The Hallmark, GO, and KEGG enrichment annotation on marker genes of the patterns V1-V6.

To explore the malignant gene patterns of squamous cancer cells, we studied 63 squamous cancer cell lines from a Sanger cell line Affymetrix gene expression project (GSE68950). These squamous cancer cell lines are derived from different parts of the body, including head and neck, lung, esophagus, cervix, and breast (Supplementary Table S2). We applied nonnegative matrix factorization (NMF) (Brunet et al., 2004) to identify molecular patterns of the cell line gene expression matrix. The algorithm recognized six patterns (V1-V6) across 63 cell lines, with the consensus matrix shown in Figure 1 A. We used Kim and Park’s gene scoring schema (Kim and Park, 2007) to extract the most relevant genes for each pattern (Supplementary Table S3). Enrichment analysis of these marker genes reflects the biological function of the pattern they belong to. As shown in Figure 1B, V1 contains many epithelial-mesenchymal transition (EMT) markers and it is also linked to other terms known to facilitate cancer progression, such as inflammatory response, hypoxia, positive regulation of cell migration, and angiogenesis. V2 is mainly associated with several growth and synthesis functions (Figure 1C); V3 is mainly related to metabolic functions (Figure 1D); V4 reflects cancer cell response to IFN-γ and TNF-α (Figure 1E); V5 seems to participate in multiple morphogenesis and cell differentiation processes (Figure 1F); and V6 restricts G protein-coupled receptor signaling pathway and regulates cell adhesion (Figure 1G). It is worth noting that the enrichment analysis was based on prior curated gene sets with known functions (KEGG, GO and HALLMARK), while the NMF patterns were identified using matrix factorization without reference. Therefore, the function of a pattern is likely to be more complicated than a GO or KEGG term, and it is difficult to summarize a pattern using one known term.

### Stromal expressed malignant patterns contribute to HNSCC subtype classification

**FIGURE 2:**
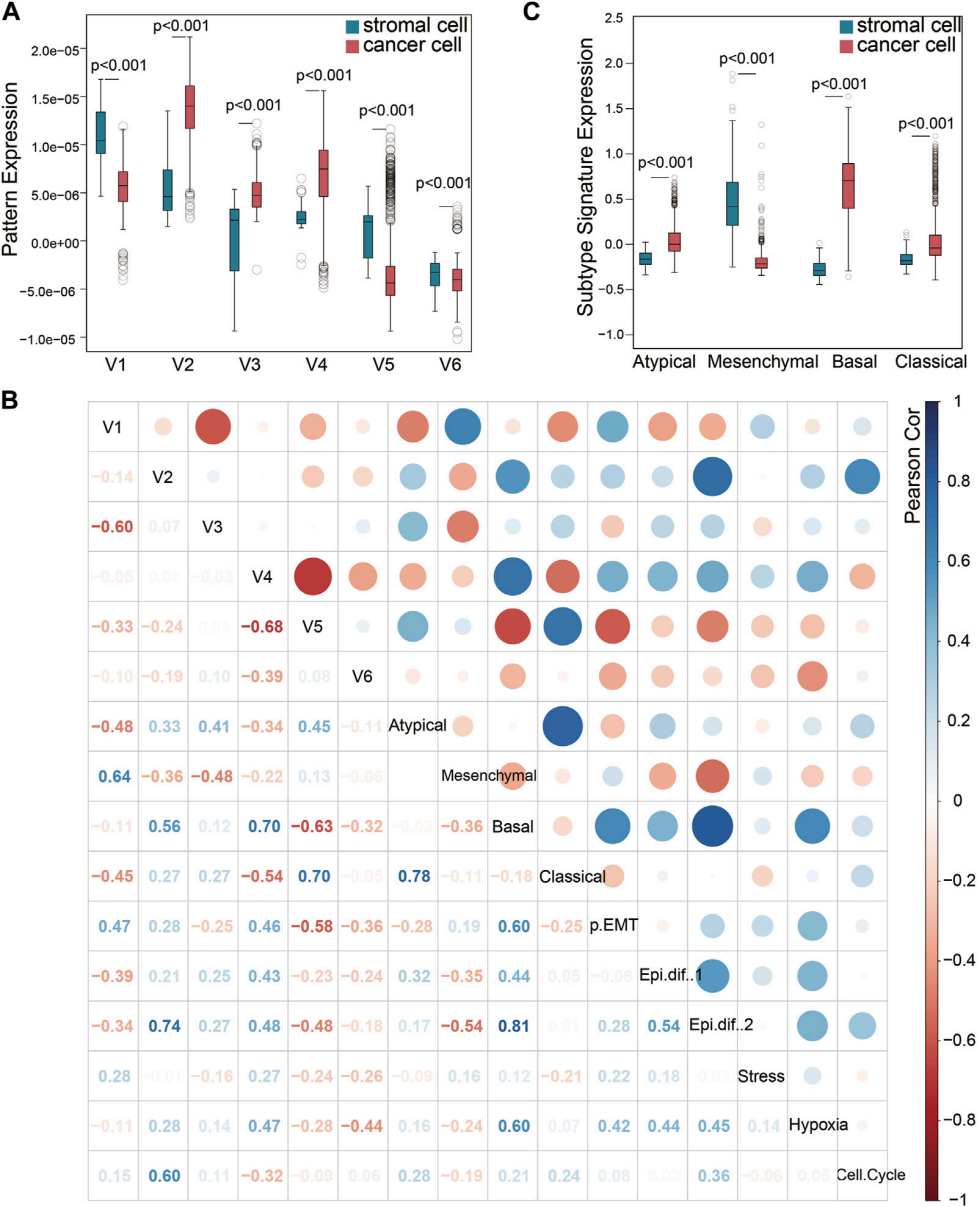
Pattern exploration in single-cell transcriptomes. **(A)** Pattens expressions in cancer cells and stromal cells. **(B)** Pearson correlation of the NMF patterns and previous signatures that has been broadly used. **(C)** The previous HNSCC subtype signature expression in cancer cells and stromal cells.

Although the six squamous malignant patterns were identified in cancer cell lines, they may be shared by other non-malignant cells in TME. The cancer-associated stromal cells in TME share multiple features with the cancer cells in solid tumors, many of which have been proven to affect prognosis. To illustrate the distribution of the 6 NMF patterns among different cell types in squamous tumors, we projected the patterns to a single-cell RNA sequencing (scRNA-seq) dataset (Puram et al., 2017) using transfer learning (Sharma et al., 2020). The scRNA-seq dataset includes 5,902 cells from 18 head and neck squamous (HNSCC) tumors after initial quality controls. We selected 1,136 cancer cells and 113 stromal cells with all six projection *p* values less than 0.05. As shown in Figure 2A, patterns V2, V3, and V4 are mainly expressed by cancer cells, while V1 and V5 are mainly expressed by stromal cells. Although the distribution of V6 is more inclined to stromal cells, the difference is not as big as the other five patterns showed.

Puram et al. have previously identified six meta-signatures (partial EMT, Epithelial differentiation one and 2, Stress, Hypoxia, and Cell cycle) that reflect common expression programs of malignant cells in this HNSCC scRNA-seq dataset (Puram et al., 2017). In addition, the TCGA HNSCC subtypes (Cancer Genome Atlas Network, 2015) (Atypical, Mesenchymal, Basal, and Classical) derived from bulk tumor sequencing were also projected to this single-cell dataset. It is worth noting that the Mesenchymal subtype has been validated as a non-malignant cell contributed signature in bulk tumor sequencing, rather than an independent cancer cell subtype. The other three subtypes have been validated by scRNA-seq (Puram et al., 2017). We evaluated the correlation of V1-V6 with these two groups of signatures in the 1,136 cancer cells and 113 stromal cells (Figure 2B). Pattern V1 was correlated to the partial EMT (p-EMT) signature (Figure 2B), which was identified as an independent predictor of nodal metastasis, grade, and adverse pathologic features (Puram et al., 2017). The marker genes of pattern V1 such as SERPINE1, ITGA5, TNC, EMP3, VIM, FN1 and THBS2 are also key genes related to p-EMT. However, pattern V1 could not be caused by EMT process because it was expressed in mesenchymal cells. This is also the key difference between pattern V1 and the p-EMT signature expressed by cancer cells. Pattern V1 also showed a high correlation with the Mesenchymal subtype signature, which is consistent with the high expression in stromal cells mentioned in Figure 2A. Therefore, we would like to explain V1 as a stromal-dominated pattern that coincides with the expression of EMT markers (Vellinga et al., 2016).

However, the other stromal-dominated pattern V5 showed almost no correlation with the Mesenchymal subtype signature, but a strong correlation (cor = 0.70) with the Classical subtype signature and a moderate correlation (cor = 0.45) with the Atypical subtype signature. It indicated that the Mesenchymal subtype signature did not capture all the features expressed by the stroma, and the cancer-associated stromal cells also contributed to the Classical and Atypical subtype signal from bulk tissue sequencing. As shown in Figure 2C, the stromal cells expressed an extremely low Basal signal but a relatively higher Atypical and Classical signal. The expression differences of Classical and Atypical signatures between the cancer cells vs. stromal cells are less than those of Basal and Mesenchymal subtypes. The correlation between Classical (Atypical) subtype signature and the stromal pattern V5 may explain this phenomenon. This finding validated that the Mesenchymal signature is derived from the stromal compartment and the Basal signature is derived from the cancer cells, while the Classical and Atypical signatures are mixed signals from both cancer cells and stromal cells.

As for cancer-dominated patterns, V2 and V4 were correlated to Basal subtype and Epithelial differentiation signature. Pattern V2 may contribute to the cell cycle signature while pattern V4 may participate in hypoxia. Pattern V3 only showed a moderate correlation with Atypical subtype.

### Stromal expressed malignant patterns are associated with poor PFI across different cancer types

**FIGURE 3:**
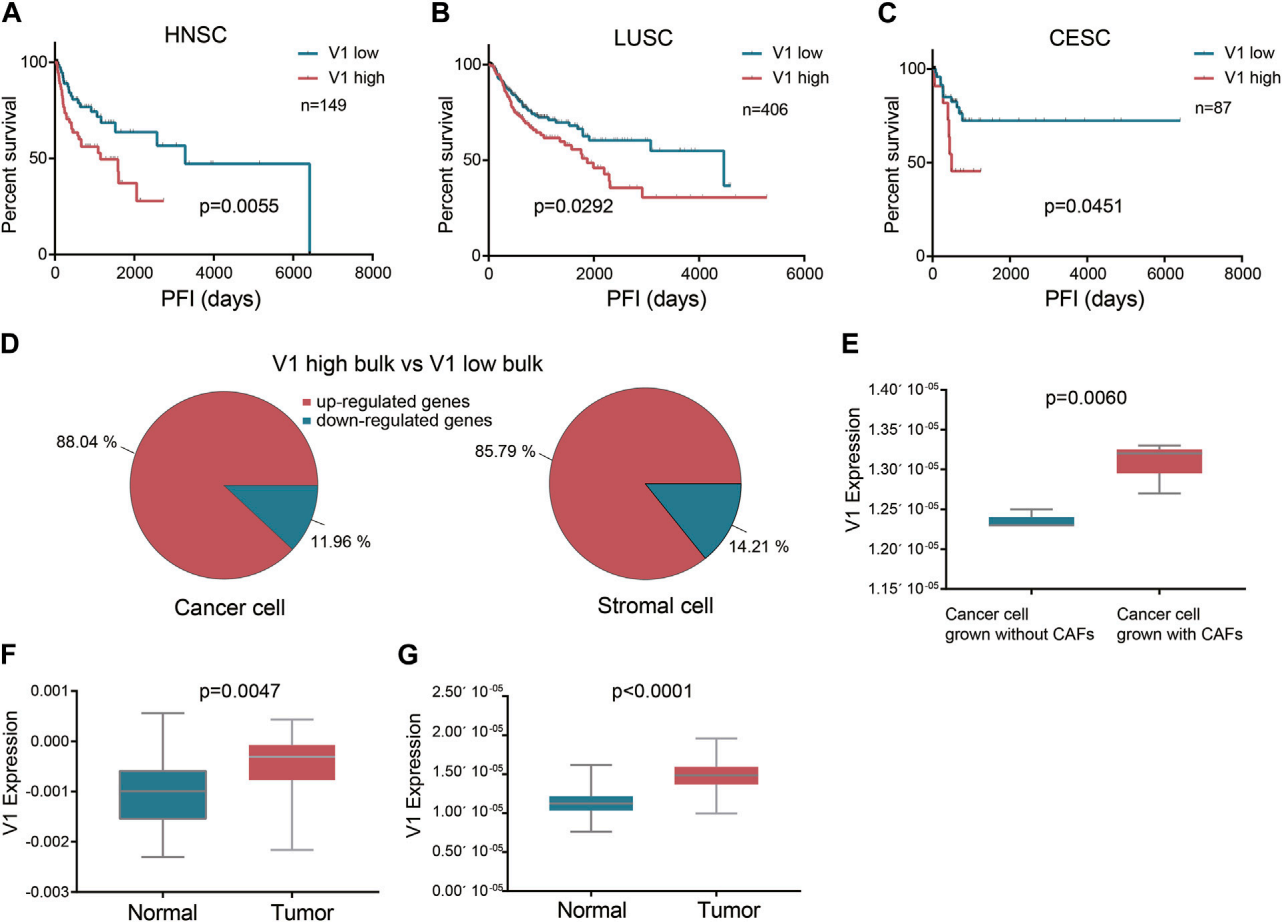
The prognostic value of the stromal expressed malignant pattern V1. **(A-C)** Survival analysis of pattern V1 in HNSCC, LUSC, and CESC patients after surgery. **(D)** The V1 marker genes expressed by each cell type between the V1 high and V1 low groups. **(E)** V1 expression in cancer cells grown without and with CAFs. **(F)** V1 expression in TCGA HNSCC normal samples and their paired tumor samples. **(G)**, V1 expression in E-MTAB-8588 HNSCC normal samples and their paired tumor samples.

To verify the influence of these squamous associated patterns on tumor progression and metastasis, we projected the six patterns to TCGA HNSCC, lung squamous cell carcinoma (LUSC), and cervical squamous cell carcinoma (CESC) datasets using transfer learning. Curated progression-free interval (PFI) recommended by the Pan-Cancer TCGA Atlas (Liu et al., 2018) was selected as the endpoint. In all the three TCGA cohorts, most patients received surgery alone before the first PFI event. In HNSCC cohort, surgery with radiotherapy was also common, while in CESC cohort, nearly half of the patients received surgery with radiochemotherapy. We found that high expression of pattern V1 was associated with significantly poor PFI in patients who received surgery alone before the first PFI event, and this phenomenon was observed in all the three squamous cancer types (Figures 3A–C).

Although pattern V1 was mainly expressed by stromal cells (mainly fibroblast), we also explored whether the high V1 expression observed in poor PFI tumors was attributed to stromal cells or cancer cells, or both. To achieve this goal, we deconvoluted the TCGA HNSCC bulk sequencing profiles to obtain the cell type transcription profiles. We divided tumors into V1 high and V1 low groups according to V1 expression in bulk tissue sequencing. Then we compared the V1 marker genes expressed by each cell type between the V1 high and V1 low groups. The result showed that in V1 high bulk tumors, both the stromal cells and the cancer cells expressed higher V1 marker genes than those in V1 low bulk tumors (Figure 3D). This result also indicated that the V1 expression in cancer cells and stromal cells might be interactive. To further verify this finding, we projected pattern V1 to five HNSCC cell lines grown without and with patient-matched cancer-associated fibroblasts (CAFs) (GSE178153). All the five cell lines showed higher V1 expression grown with CAFs than grown without CAFs (Figure 3E), indicating that pattern V1 expressed in cancer cells could be induced by CAFs. Although pattern V1 is mainly expressed by non-malignant cells, it does play a role in tumor malignancy. Evidence to this hypothesis is that the normal tissue in TCGA HNSCC dataset expressed lower V1 compared to their paired tumor (*p* = 0.0047, Figure 3F). We observed the same phenomenon in another cohort (E-MTAB-8588) with 98 paired normal and HNSCC tumor samples (*p* < 0.0001, Figure 3G).

In the TCGA LUSC cohort, most patients received surgery alone before the PFI event, while in the HNSCC cohort, surgery with radiotherapy is quite common. We selected 80 HNSCC patients who received surgery (margin negative) and full course adjunctive radiotherapy before the PFI event to perform the univariate cox analysis. The result showed that V1 expression had no influence on the PFI after surgery plus radiotherapy (*p* = 0.3073). However, when we used a well-validated radiosensitivity score RSI (Eschrich et al., 2009) to divide those patients into radiosensitive (RS) and radioresistant (RR) groups, we found in the RS group V1 expression did not affect the PFI, while in the RR group high V1 was related to worse PFI after surgery plus radiotherapy, although with a *p*-value of 0.0532. Therefore we assume that SCCs with high V1 expression show a high tendency to recurrence and metastasis even after surgery with a negative margin, but radiation delivered to the tumor bed and high-risk areas after surgery may weaken the effect of high V1, depending on the tumor radiosensitivity. We did not further investigate the effect of V1 on PFI after chemotherapy because there was large heterogeneity in the application of chemotherapy, and in most cases, chemotherapy was implemented with radiotherapy simultaneously.

**FIGURE 4:**
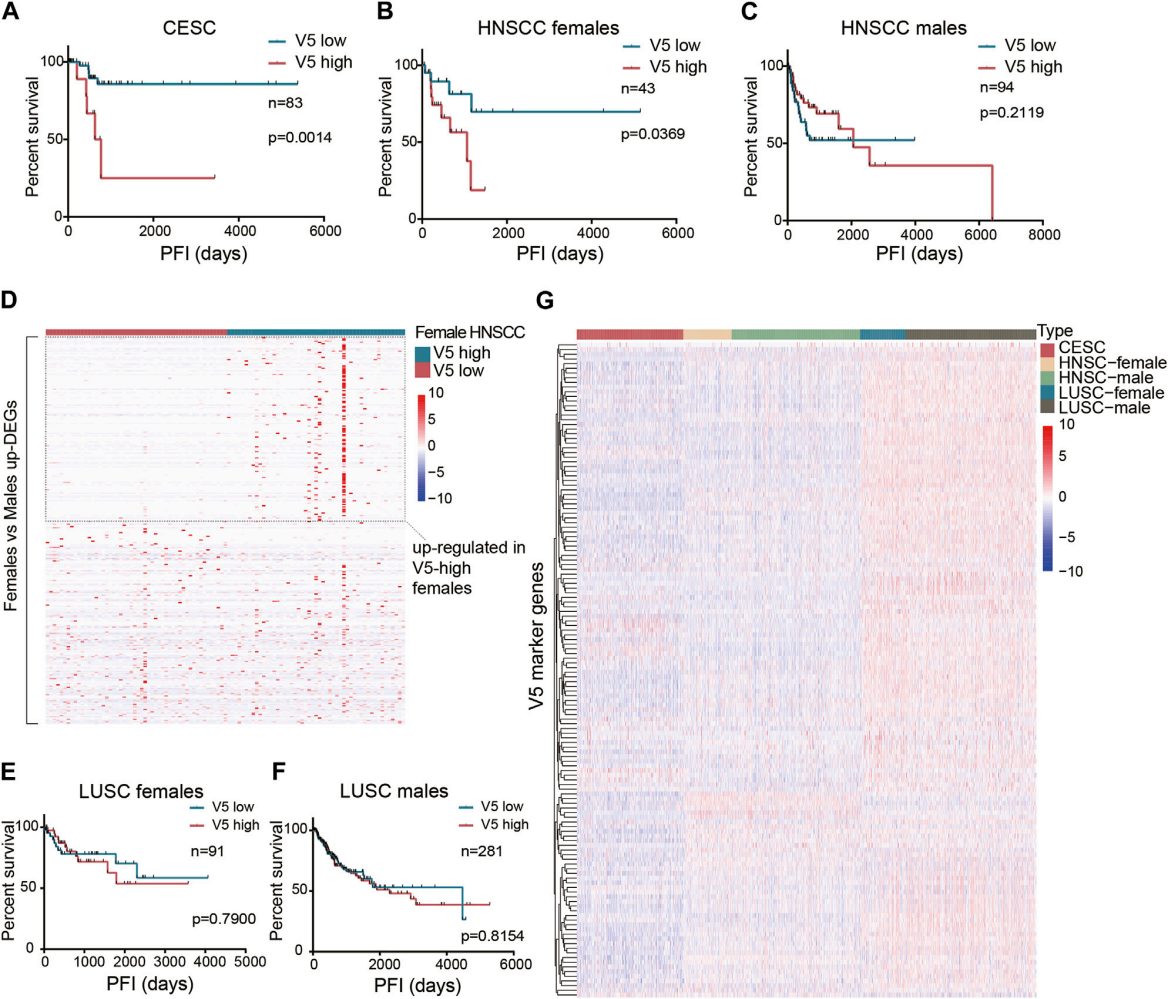
The prognostic value of the stromal expressed malignant pattern V5. **(A)** Survival analysis of pattern V5 in CESC patients after surgery. **(B-C)** Survival analysis of pattern V5 in HNSCC females and males after surgery. **(D)** Female-upregulated genes expression in V5-high and V5-low female HNSCCs. **(E-F)** Survival analysis of pattern V5 in LUSC females and males after surgery. **(G)** V5 marker genes expression in different cancer types.

Unlike the general prognostic value of pattern V1, we found that pattern V5 affected the PFI after surgery only in the CESC dataset (Figure 4A). Since CESC is special because it is female-only, we continued to study whether gender limits the prognostic value of pattern V5. After setting gender subgroups, we re-examined the effect of V5 on PFI after surgery (median V5 was used as cutoff). Interestingly, higher V5 did show a worse PFI in HNSCC females (*p* = 0.0369, Figure 4B) rather than in males (Figure 4C). In HNSCC males, the curves even showed an opposite tendency. The reason why V5 showed such a different effect on different sex was not clear. But we did identify 315 upregulated genes in female HNSCCs compared with male HNSCCs (FDR<0.05, fold change>2), and half of them (152 in 315) were also upregulated in high V5 females compared with low V5 females (Figure 4D), which partially explains the gender bias of high-V5 effect. However, in the LUSC dataset, we did not observe a significant difference (Figures 4E,F): the survival curves of high V5 and low V5 showed a crossover in females. As shown in Figure 4G, the overall expression of V5 marker genes in LUSC is quite different from that in HNSCC and CESC, indicating an expression-dependent cancer-type specificity of V5’s prognostic ability.

## Discussion

The study aimed to explore the malignant role of stroma in SCCs across different sites. We choose NMF for pattern recognition not only because of its excellent performance on the transcriptome but also because it considers all genes in the transcriptome without any reference or functional annotation. The algorithm always finds the most significant patterns and makes sure the combination of the patterns accurately reflects the target. That explains why the NMF algorithm often identifies cell type features when it is run in bulk sequencing data, since cell type usually contributes to the most significant differences within tumor tissue. To get the gene patterns reflecting the biological characteristics at the cell level, we performed NMF in sequencing data of the same cell type (cell line RNA-seq). Since NMF may not recognize malignant patterns from numerous non-malignant features in stromal cells and cancer stroma shares many genetic features with cancer cells, we performed NMF in squamous cancer cell lines and proved that two malignant patterns are mainly expressed in cancer stroma.

The four-subtype classification of HNSCC based on bulk analyses (Atypical, Mesenchymal, Basal, and Classical) had been widely accepted until Puram et al. proved that no malignant cells in a scRNA-seq dataset mapped to the Mesenchymal subtype. They found the Mesenchymal signature in bulk sequencing was actually a stromal signal coming from the Basal subtype tumors. They also validated that the malignant cells of each tumor did map exclusively to one of the other three subtypes (Atypical, Basal, and Classical), even after control for TME. However, there have been no signatures describing the stromal composition of the Atypical and Classical bulk subtypes. In this study, we found a stroma-dominated pattern V5 showing a high correlation with the Classical signature, moderate correlation with the Atypical signature, but no correlation with the Mesenchymal signature (Figure 2B). This pattern reflected a new malignant feature expressed by stromal cells of Classical and Atypical HNSCC tumors. Our results validated that the HNSCC Mesenchymal signature is derived from the stromal compartment and the Basal signature is derived from the cancer cells, while the Classical and Atypical signatures are mixed signals from both cancer and stromal cells. In TCGA CESC and HNSCC females, high expression of pattern V5 was associated with poor PFI. Further study may focus on the gender-biased prognostic mechanism of pattern V5.

Another stroma-dominated pattern V1 is associated with poor PFI across different cancer types (HNSCC, LUSC, and CESC). The marker genes of pattern V1 were enriched in the Hallmark EMT gene set. However, we believe this change is unrelated to EMT because V1 was mainly expressed by mesenchymal cells. In fact, researches on colorectal and urothelial cancers have shown that stromal cells are a major source of EMT-related gene expression in bulk transcriptomes, rather than epithelial-derived cancer cells (Isella et al., 2015; Wang et al., 2018; Li et al., 2023; Cañellas-Socias et al., 2024), which seems contrary to the biological concept of EMT. This phenomenon could be explained by an activated stromal state that coincides with cancer cell expressed EMT-related markers (Vellinga et al., 2016). Although these genes are clearly expressed in cancer cells after EMT, the expression level was far lower than that in CAFs. In addition, we found that V1 expression in cancer cells could be induced by co-culturing with CAFs. A similar phenomenon has been found in colorectal cancer that cancer cells express mesenchymal genes in a high-stroma context (Vellinga et al., 2016). Pattern V1 demonstrates the key effect of cancer stroma on the progression and metastasis of different types of squamous cell carcinoma. Interestingly, radiation delivered to the tumor bed and high-risk areas after surgery may weaken the effect of high V1 on recurrence and metastasis, depending on the tumor radiosensitivity. Further study may compare the different outcomes of surgery alone and surgery plus radiotherapy in radiosensitive tumors with high and low V1 expression, respectively. The pattern V1 may bring a new indication for postoperative radiotherapy.

Previous theories have been considering cancer stroma as “dangerous liaisons” interacting with cancer cells and other components in TME to form a cancer-supportive environment (Chen and Song, 2019; Gieniec et al., 2019). In this study, we confirmed the prognostic value of stromal gene features and its universality in SCCs. Therefore, We suggest that the tumor genetic subtype classification may be changed to a new system that integrates both malignant and non-malignant components. There are potential limitations to this work, given the lack of validation in cell experiments. Adding this part of the experimental content in the future may provide evidence for the application of this research in the clinical field.

## Conclusion

In this study, we confirmed the prognostic value of stromal gene features and its universality. Therefore, we suggest that the tumor genetic subtype classification may be changed to a new system that integrates both malignant and non-malignant components.

## Data Availability

The datasets presented in this study can be found in online repositories. The names of the repository/repositories and accession number(s) can be found in the article/Supplementary Material.

## Supplementary material

SUPPLEMENTARY TABLE S1 A total of 303 GEO DNA methylation profiles of known cell types as reference for deconvolution.

SUPPLEMENTARY TABLE S2 Squamous cancer cell lines from the Sanger cell line Affymetrix gene expression project (GSE68950). These squamous cancer cell lines are derived from different parts of the body, including head and neck, lung, esophagus, cervix, and breast.

SUPPLEMENTARY TABLE S3 Marker genes of each pattern identified by NMF.

SUPPLEMENTARY FIGURE S1 Deconvolution of the TCGA HNSCC dataset.

SUPPLEMENTARY FILE S1 Details of data analysis.
