# Supplementary figures and images for: Stromal cell-expressed malignant gene patterns contribute to the progression of squamous cell carcinomas across different sites
Source: Front Genet. 2024 Jul 12;15:1342306. doi: 10.3389/fgene.2024.1342306 (PMC11272565; doi:10.3389/fgene.2024.1342306)

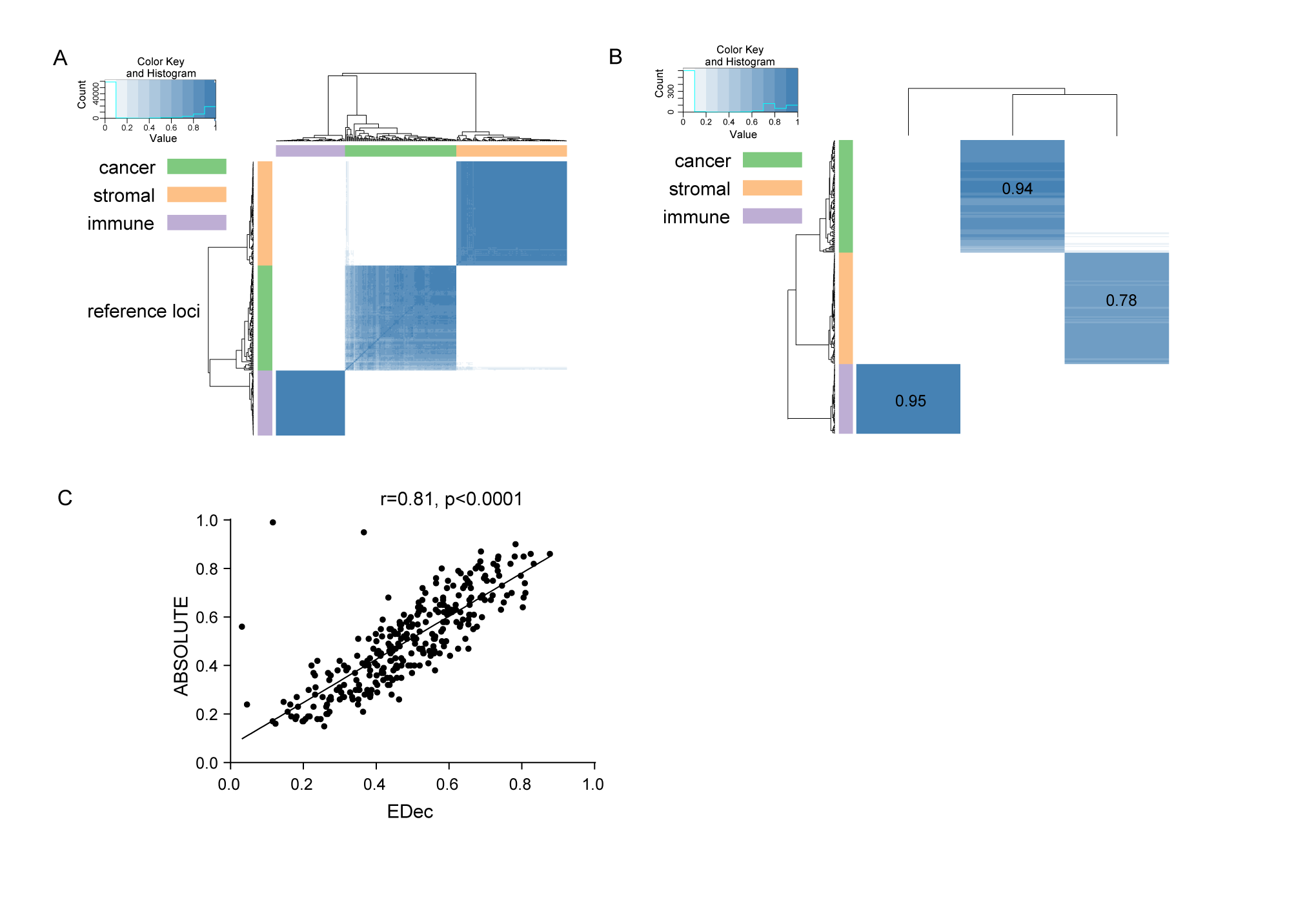

Supplement: Supplementary file 3 [file Image1.TIF]
