# Supplementary material for: Stromal cell-expressed malignant gene patterns contribute to the progression of squamous cell carcinomas across different sites
Source: Front Genet. 2024 Jul 12;15:1342306. doi: 10.3389/fgene.2024.1342306 (PMC11272565; doi:10.3389/fgene.2024.1342306)
Supplement: Supplementary file 5 [file Table4.DOCX]

The datasets used in this study were all available as described in Methods and Supplementary files. The analysis detail of these data is described as below:

1. **Pattern recognition:**

The microarray gene expression profiles of the 63 squamous cancer cell lines were selected from the Sanger Cell Line Affymetrix Gene Expression Project (Supplementary table 1, <https://www.ncbi.nlm.nih.gov/geo/query/acc.cgi?acc=GSE68950>).

R package “NMF” ^1^was performed on the 63 squamous cancer cell lines for pattern recognition: We firstly estimate the optimal rank number by performing *NMF::nmf(data, 2:8, nrun=40, seed=123456, .opt='vP')*. Then using rank=6 we performed the NMF for nrun=200. Marker genes of each pattern was selected using the *extractFeatures* function of the NMF package.

1. **Transfer learning:**

We projected the patterns to specific datasets (the HNSCC scRNA-seq dataset^2^, the HNSCC cell lines with CAFs dataset^3^, the TCGA HNSCC, CESC, and LUSC bulk sequencing dataset <https://portal.gdc.cancer.gov/>) to explore the relationship between gene patterns and phenotypes. This transfer learning process was performed using the R package “projectR” ^4^ with default parameters. The patterns x genes matrix from the NMF results was project to the object matrix of genes x samples to get the patterns x samples matrix. Projection with P values less than 0.05 was select for further exploration.

1. **Deconvolution:**

The Edec R package^5^ was used for deconvolution of the TCGA bulk sequencing data. A total of 303 GEO DNA methylation profiles of known cell types (cancer cells, stromal cells, and immune cells, Supplementary Table 3) were collected as reference^6^.

First, we identified 400 DNA loci from the 450 k methylation profile which allowed us to accurately distinguish the three reference cell types:

EDec::run_edec_stage_0(reference_meth,

reference_classes,

max_p_value = 1e-10,

num_markers = 400,

version = "one.vs.rest")

Based on the 400 loci, we deconvoluted the TCGA HNSCC methylation profile into 3 subtypes:

EDec::run_edec_stage_1(meth_bulk_samples,

informative_loci,

num_cell_types = 3)

The 3 parts showed a high correlation with cancer, stroma, and immune reference profiles, respectively (Supplementary Figure 1B). Then we estimated the cell type gene expression through a constrained least squares fit^5^ based on the cell type proportions obtained by deconvolution:

EDec::run_edec_stage_2(gene_exp_bulk_samples, cell_type_props)

**Refs:**

1. Gaujoux R, Seoighe C. A flexible R package for nonnegative matrix factorization. *BMC bioinformatics.* 2010;11:367.

2. Puram SV, Tirosh I, Parikh AS, et al. Single-Cell Transcriptomic Analysis of Primary and Metastatic Tumor Ecosystems in Head and Neck Cancer. *Cell.* 2017;171(7):1611-1624.e1624.

3. Wiechec E, Magan M, Matic N, et al. Cancer-Associated Fibroblasts Modulate Transcriptional Signatures Involved in Proliferation, Differentiation and Metastasis in Head and Neck Squamous Cell Carcinoma. *Cancers.* 2021;13(13).

4. Sharma G, Colantuoni C, Goff LA, Fertig EJ, Stein-O'Brien G. projectR: an R/Bioconductor package for transfer learning via PCA, NMF, correlation and clustering. *Bioinformatics (Oxford, England).* 2020;36(11):3592-3593.

5. Onuchic V, Hartmaier RJ, Boone DN, et al. Epigenomic Deconvolution of Breast Tumors Reveals Metabolic Coupling between Constituent Cell Types. *Cell reports.* 2016;17(8):2075-2086.

6. Li G, Jiang Y, Li G, Qiao Q. Comprehensive analysis of radiosensitivity in head and neck squamous cell carcinoma. *Radiother Oncol.* 2021;159:126-135.
